# Cerebral air embolism as a complication of peptic ulcer in the gastric tube: case report

**DOI:** 10.1186/1471-230X-11-139

**Published:** 2011-12-21

**Authors:** Takahisa Suzuki, Takafumi Ando, Akihisa Usami, Masataka Shinoda, Hitomi Takashi, Mutsumi Murayama, Isako Uchiyama, Kazuhiro Morise, Shinya Endo, Nobuhiro Haruki, Kazuhiro Tashiro, Hidemi Goto

**Affiliations:** 1 Department of Gastroenterology, Nagoya University Graduate School of Medicine. (65 Tsurumai-cho, Showa-ku), Nagoya City, Aichi, (466-8550), Japan; 2 Department of Gastroenterology, Toyota Memorial Hospital. (1-1, Heiwa-cho), Toyota City, (471-8513), Japan; 3 Department of Surgery, Toyota Memorial Hospital. (1-1, Heiwa-cho), Toyota City, (471-8513), Japan; 4 Department of Pathology, Toyota Memorial Hospital. (1-1, Heiwa-cho), Toyota City, (471-8513), Japan

## Abstract

**Background:**

The reported incidence of ulcer formation in the gastric tube in esophageal replacement is rare.

**Case Presentation:**

This is the first report of a case of cerebral air embolism as a result of spontaneous perforation of an ulcer in the constructed gastric tube into the pulmonary vein during post-operative follow-up in a patient with esophageal cancer.

**Conclusions:**

Cerebral air embolism is a rare complication of penetrating gastric ulcer, but should be considered in patients with a history of esophagectomy with gastric conduit that present with acute neurologic findings.

## Background

The reported incidence of ulcer formation in the gastric tube in esophageal replacement ranges from 2.6%-19.4% [1,2]. Here, we describe the first report of a case of cerebral air embolism as a result of spontaneous perforation of a gastric tube ulcer into the pulmonary vein. This case highlights the importance of ongoing awareness of the risk of recurrence of peptic ulcers in the constructed gastric tube during post-operative follow-up in patients with esophageal cancer.

## Case presentation

**Figure 1:**
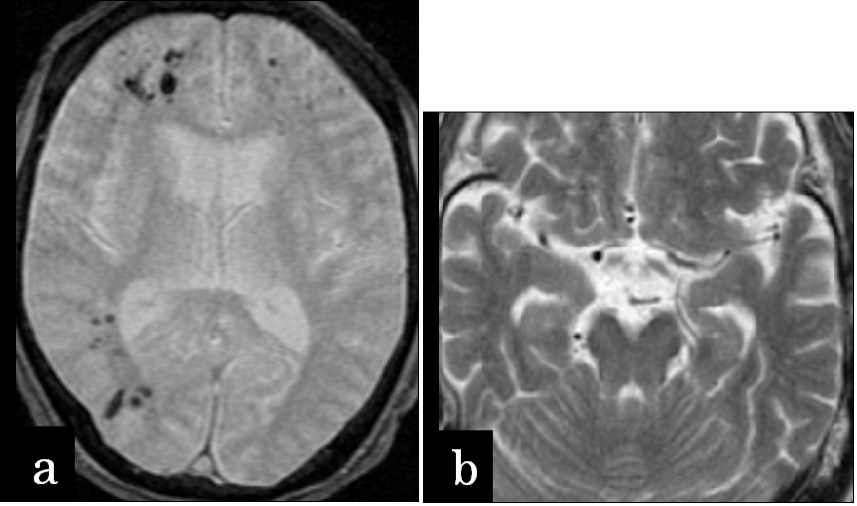
**Cranial MRI (T2)**. Small collections of gas were revealed within the right hemisphere and frontal lobe (a) and internal carotid artery siphon (b).

**Figure 2:**
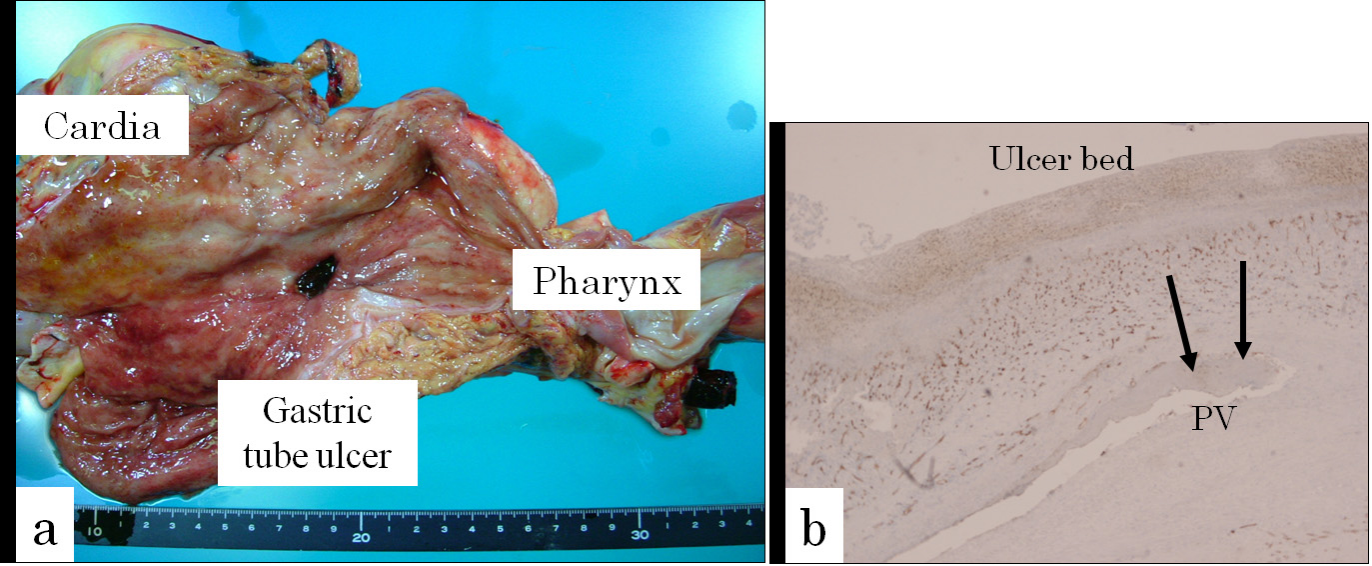
**Postmortem specimen of the gastric tube**. a: Autopsy showed an ulcer of 1.5 cm diameter in the middle of the gastric tube. b: Immunostaining with CD34 showed complete loss of endothelial cell structure of the branch of the pulmonary vein beneath the ulcer base of the gastric tube (arrows), indicating perforation of the vessel.

A 68-year-old man who had undergone subtotal esophagectomy for squamous cell carcinoma of the lower thoracic esophagus eight years previously was admitted with high fever and right hemiplegia. On examination, body temperature was elevated at 40.1°C, heart rate was 126 beats/min, and blood pressure was 170/110 mmHg. The abdomen was soft and flat, and no tenderness was observed. Antibiotic treatment was started. Immediately after drinking a glass of water at 3 h after admission, the patient suddenly developed respiratory distress and lost consciousness. He remained unresponsive and developed a flaccid paralysis. Cranial computed tomography (CT) demonstrated small collections of gas within the right hemisphere and frontal lobe, while cranial magnetic resonance imaging (MRI) revealed small collections of gas within the right hemisphere and frontal lobe (Figure 1a) and internal carotid artery siphon (Figure 1b). Contrast-enhanced thoraco-abdominal CT showed wall thickening and emphysema of the gastric tube, but no metastatic lesions were found in the mediastinum. The patient was diagnosed with cerebral air embolism as a complication of a gastric tube disorder. Despite supportive therapy, his status continued to deteriorate, and he was declared dead 13 days later. Autopsy showed an ulcer of 1.5 cm diameter in the middle of the gastric tube which had caused complete rupture down to the muscularis propria (Figure 2a). Immunostaining with CD34 showed complete loss of the endothelial cell structure of the branch of the pulmonary vein beneath the ulcer base of the gastric tube, indicating perforation of the vessel (Figure 2b). No evidence of *Helicobacter pylori *infection was seen.

Several etiological mechanisms for the formation of gastric tube ulcers have been postulated, including hypersecretion of gastric juice, inadequate drainage from the pylorus, and breakdown of the mucous and mucosal barrier as a result of disturbed vascular circulation of the gastric tube [3]. In our case, gastric pedicle stasis in the eight years after surgery due to denervation or the gastric tube itself may have caused delayed gastric emptying, with a resulting increase in gastrin secretion and high acidity. Peptic ulcer of the gastric tube may penetrate into any adjacent organ [4], including the right pleural cavity [5], bronchi [6], pericardial cavity [7], thoracic aorta [8], pulmonary artery [6], left brachiocephalic vein [9], and sternum [2]. Cerebral air emboli are usually caused by trauma or an invasive procedure, including upper gastrointestinal endoscopy [10], affecting one of the blood vessels. In the present case, however, the patient had no trauma, invasive procedures, or central line access. To our knowledge, this is the first reported case of cerebral air embolism resulting from spontaneous perforation of a gastric tube ulcer into the pulmonary vein.

## Conclusions

Cerebral air embolism is a rare complication of penetrating gastric ulcer, but should be considered in patients with a history of esophagectomy with gastric conduit that present with acute neurologic findings. Postoperative endoscopic surveillance should be considered based on the risk-benefit profile and clinical symptoms of each individual patient.

### Consent

Written informed consent was obtained from the patient's relatives for publication of this case report.

## Competing interests

The authors declare that they have no competing interests.

## Authors' contributions

TS, AU, MS, HT, MM, IU, KM, SE, NH, and KT have made substantial contributions to acquisition of data and interpretation of data. TS, TA, MS, and HG have been involved in drafting the manuscript or revising it critically for important intellectual content. TA and HG have given final approval of the version to be published. All authors read and approved the final manuscript.

## Authors' information

All authors are specialized in diagnoses and treatments for all diseases occurring from both upper and lower gastrointestinal tract (esophagus, stomach, intestine, colon). TA and HG also are working on clinical and basic research for all diseases occurring from both upper and lower gastrointestinal tract. Research projects of TA and HG include Clinical and Basic Research for advanced endoscopic therapy, Clinical Research for Ultra-Zoom Endoscopy, Molecular Biological Research on GISTs, Clinical Research for EUS-Elastography, *Helicobacter pylori *Infection and Gastric cancer, Gastroesophageal reflux disease, Barrett's esophagus, and Esophageal adenocarcinoma.

## Pre-publication history

The pre-publication history for this paper can be accessed here:

http://www.biomedcentral.com/1471-230X/11/139/prepub
